# A high-throughput microscopy method for single-cell analysis of event-time correlations in nanoparticle-induced cell death

**DOI:** 10.1038/s42003-019-0282-0

**Published:** 2019-01-24

**Authors:** Alexandra Murschhauser, Peter J. F. Röttgermann, Daniel Woschée, Martina F. Ober, Yan Yan, Kenneth A. Dawson, Joachim O. Rädler

**Affiliations:** 1 Faculty of Physics and Center for NanoSciene (CeNS), Ludwig-Maximilians-Universität, Geschwister-Scholl-Platz 1, Munich, 80539 Germany; 2 Centre for BioNano Interactions, School of Chemistry and Chemical Biology, University College Dublin, Belfield, Dublin 4, Ireland

## Abstract

The temporal context of cell death decisions remains generally hidden in ensemble measurements with endpoint readouts. Here, we describe a method to extract event times from fluorescence time traces of cell death-related markers in automated live-cell imaging on single-cell arrays (LISCA) using epithelial A549 lung and Huh7 liver cancer cells as a model system. In pairwise marker combinations, we assess the chronological sequence and delay times of the events lysosomal membrane permeabilization, mitochondrial outer membrane permeabilization and oxidative burst after exposure to 58 nm amino-functionalized polystyrene nanoparticles (PS-NH_2_ nanoparticles). From two-dimensional event-time scatter plots we infer a lysosomal signal pathway at a low dose of nanoparticles (25 µg mL^−1^) for both cell lines, while at a higher dose (100 µg mL^−1^) a mitochondrial pathway coexists in A549 cells, but not in Huh7. In general, event-time correlations provide detailed insights into heterogeneity and interdependencies in signal transmission pathways.

## Abstract (web summary)

Alexandra Murschhauser et al. present a high-throughput microscopy method for the analysis of single-cell longitudinal data using fluorescent markers. They apply the method to cancer cell lines exposed to cytotoxic nanoparticles and find that 58 nm PS-NH_2_ nanoparticles induce a lysosomal pathway and, at high doses, lead to cell death via a mitochondrial pathway.

## Introduction

The signaling pathways triggered by interactions between nanomaterials and living cells have raised many unexpected questions^1^. The study of adverse effects of nanoparticles is complicated by the fact that cells respond to these agents in a highly heterogeneous way. Various scenarios of apoptotic or necrotic pathways and cross talk thereof are likely to exist. Moreover, the impact of nanoparticles compared to drugs is prone to considerable cell-to-cell variations in the timelines and points of time, when cell death occurs. Nanoparticle-cell interactions exhibit varying, stochastic impacts on intracellular and extracellular signaling and become temporally disordered in the course of their progression. Particle contact with the cell surface, and trafficking along and across it occur at diverse time points following application^2^. Upon entry, particles may even be conveyed to different intracellular locations in different cells along pathways, which, although similar to each other in sequence, may be temporally shifted in relation to each other. Indeed, this temporal heterogeneity can result in divergent particle residence times within specific organelles or regions of the cell, and lead to qualitatively distinct event sequences in different cells that correspond to different signal transduction mechanisms. Taking amino-modified nanoparticles as an example, the analysis of temporal correlations between steps in signaling cascades within a cell population clearly points to different sequences of events and the engagement of multiple apoptotic pathways involving both lysosomes and mitochondria^3,4^.

Cationic, amino-modified polystyrene nanoparticles (PS-NH_2_ nanoparticles) are interesting examples since they exhibit clear cytotoxicity^5–8^. Consequently, they have been considered as a model system, and previous studies have yielded some insight into the pertinent mechanisms. It is currently assumed that protonation of amino groups in the acidic environment of lysosomes results in lysosomal swelling and ultimately leads to lysosomal rupture and particle flux into the cytosol^9^. However, the cellular pathways that are activated further downstream and finally trigger cell death are still poorly understood. Previous work employing high content analysis suggested that 58 nm PS-NH_2_ nanoparticles trigger apoptosis via the lysosomal pathway^3,4^. Dose-response curves indicated that lysosomal membrane permeabilization (LMP) is likely to precede permeabilization of the outer mitochondrial membrane (MOMP). Both LMP^6,10^ and MOMP^11–13^ are key events in programmed cell death and are partially interdependent, which suggests a degree of lysosomal-mitochondrial cross talk^14–16^. The destabilization of lysosomes due to nanoparticle accumulation brings about the release of cathepsin-D, which in turn induces apoptosis via the intrinsic mitochondrial pathway^3,17–19^. The breakdown of mitochondria itself leads to the release of cytochrome C and an abrupt rise in levels of cytosolic reactive oxygen species (ROS)^20^. At the point of no return, activation of the effector caspases 3 and 7 initiates the execution pathway, which becomes manifest in the externalization of phosphatidylserine (PhS) to the outer leaflet of the plasma membrane and the loss of plasma membrane integrity^3^. As most events occur within minutes after cell treatment, it is well understood that there is a need for real-time imaging at the single-cell level^3,21–24^. Thus, only time-resolved live-cell imaging of individual cells sheds light on the heterogeneous dynamics and the order of events in the decision trees leading to programmed cell death^4,25,26^.

Here, we employ a high-throughput single-cell time-lapse microscopy on micro-arrays to analyze the sequence of appearance of cell death-related markers in human epithelial cancer cell lines A549 and Huh7. A549 cells are frequently used as a lung model system in cytotoxic nanoparticle studies, and Huh7 are suitable model cells as nanoparticles are accumulated in the liver, when taken up by the intestine. We define and assess cellular event times using automated fitting of fluorescent time traces of individual cells. When this approach is applied to pairwise combinations of different markers, event correlations are revealed at the single-cell level. Cluster analysis of the two-dimensional scatter plots then allows one to infer the order of marker events. We show that 58 nm PS-NH_2_ nanoparticles induce a lysosomal pathway in A549 cells, but we also find that at a higher nanoparticle dose a subpopulation of cells undergoes cell death mediated by a mitochondrial pathway. Such cross talk between signals induced by nanoparticles is typically not discernible in conventional average-based analyses, leading to the loss of information relevant to the biological impact of nanoparticles. We anticipate that our high-throughput assay will prove to be a powerful tool for the study of mechanistic and temporal heterogeneity in cellular responses to diverse types of perturbation.

## Results

### Automated assessment of individual timelines in single-cell arrays

**Fig. 1:**
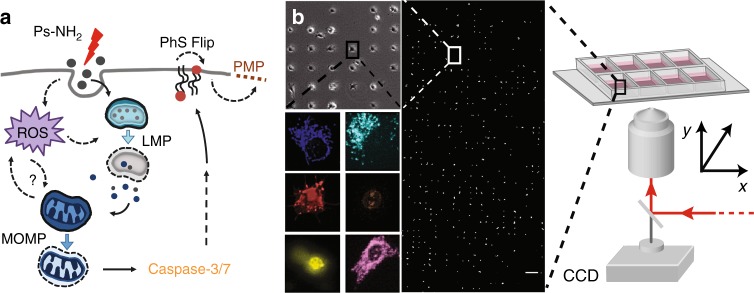
Nanoparticle-induced cell death studied by high-throughput single-cell time-lapse fluorescence microscopy. **a** Schematic illustration of the uptake and potential signaling pathways induced by exposure to 58 nm cationic polystyrene nanoparticles (PS-NH_2_ nanoparticles). Upon interaction with cells, nanoparticles can be directed into one of two interconnected pathways (i.e. LMP-dependent and MOMP-dependent) that trigger the activation of caspases 3/7, which ultimately results in the externalization of phosphatidylserine to the outer leaflet of the plasma membrane (PhS-Flip) and the permeabilization of the plasma membrane (PMP). **b** Schematic representation of the experimental single-cell platform. Cells are seeded on a micropatterned surface with one cell per square adhesion site (30 × 30 µm), and stained with a panel of fluorescence dyes to monitor key organelles and steps associated with cell death. Views of representative cells stained for mitochondria (blue), lysosomes (cyan), phosphatidylserine (red), nucleus (brown), caspase activation (yellow), and OxBurst (purple) are also shown. Scale bar: 200 µm

Six fluorescent markers, which are frequently used to study and classify forms of cell death, were utilized to track the time course of events along signaling pathways (Fig. 1a). LysoTracker was used as an indicator for lysosomal plasma permeabilization (LMP), tetramethylrhodamine methyl ester (TMRM) for MOMP, and CellROX for ROS production as well as detection of the oxidative burst (OxBurst). The activation of caspases 3 and 7 was monitored with CellEvent Caspase 3/7 (CASP-3), the externalization of PhS to the outer leaflet of the plasma membrane (PhS-Flip) was detected with pSIVA-IANBD, and propidium iodide (PI) as well as Toto-3 Iodide were used to visualize plasma membrane permeabilization (PMP). For the purpose of this study, the focus will be on the so-called early markers, which are associated with the initiation phase of cell death (LysoTracker, TMRM, and CellROX). The late markers (Caspase 3/7, pSIVA-IANBD, and PI or Toto-3 Iodide) are provided to complete the assessment of cell death indicators. As the data exhibit heterogeneity in timing and event order, we will avoid the unambiguous assignment of the proper term of cell death, in particular apoptosis versus necrosis, and leave the issue to the discussion.

Figure 1b shows the workflow used for single-cell time-lapse measurements. Single-cell arrays were fabricated by selective plasma-induced patterning (microscale plasma-initiated patterning; µPIP) of eight-well matrices with cell-repellent (polyethylene glycol) and cell-adhesive (fibronectin) sites following a previously established protocol^26,27^. A detailed description of the assay can be found in the Methods section. Briefly, cells were seeded on the micropatterned eight-well slides and left to settle and redistribute for 6 h. After slide preparation, cells were exposed to 58 nm PS-NH_2_ nanoparticles. We chose (25 µg mL^−1^) as a “low” exposure and (100 µg mL^−1^), which is considered as “high” exposure. At a concentration of 10 µg mL^−1^ nanoparticles, no considerable cell death was found (Supplementary Table 1). We also use a positive control, the cell death-inducing protein kinase inhibitor staurosporine (STS, 2 µM) and a negative control, i.e. culture medium including fluorescence markers, but no nanoparticles. It is noteworthy that before addition to the cells, nanoparticles are premixed with media supplemented with serum and hence the administered nanoparticles are decorated with a biomolecular corona. The open eight-well format allowed for preparation of eight different experimental conditions in parallel. In some experiments, the Hoechst 33342 DNA marker was added in addition. Markers and nanoparticles were applied in parallel without any further washing steps. The fluorescence markers were detected by time-lapse fluorescence microscopy at the single-cell level. Representative movies of a single-cell array stained with CellROX and PI (after cell exposure to STS), and of single-cell observations after the administration of 25 µg mL^−1^ PS-NH_2_ nanoparticles, CellROX, and LysoTracker, are provided as Supplementary Movie 1 and Supplementary Movie 2.

### Marker-specific time traces

**Fig. 2:**
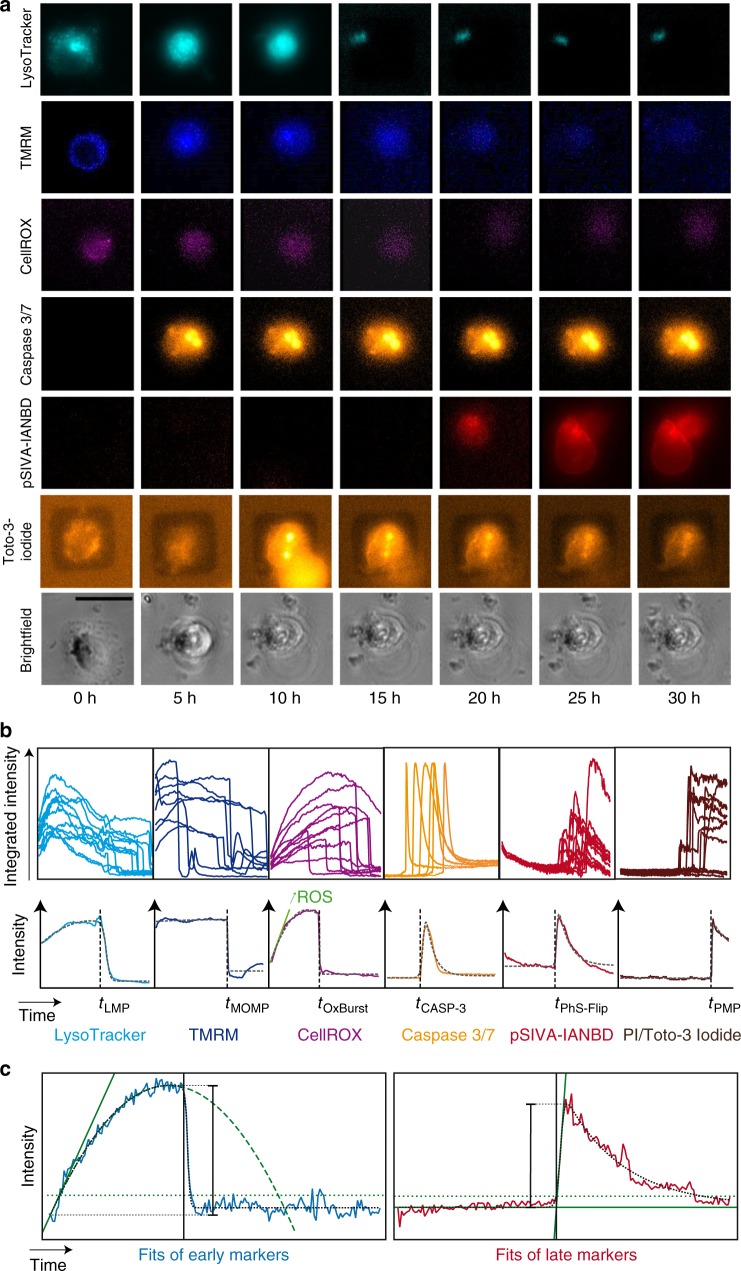
Image series and time traces of fluorescence markers. **a** Representative time-lapse images of cells that were exposed to 58 nm 100 µg mL^−1^ amino-modified polystyrene nanoparticles (PS-NH_2_ nanoparticles), imaged over 30 h, and stained for lysosomes (LysoTracker, cyan), functional mitochondria (tetramethylrhodamine methyl ester (TMRM), blue), reactive oxygen species (ROS) and oxidative burst (OxBurst; CellROX, purple), activated caspase 3/7 (Caspase 3/7, orange), and flipped phosphatidylserine (pSIVA-IANBD, red). Contrast was adjusted for better visibility. Scale bar: 30 µm. **b** Representative time traces of each marker in single cells: lysosomes (LysoTracker), MOMP (TMRM), OxBurst (CellROX), caspase 3/7 activation (Caspase 3/7 marker), PhS-Flip (pSIVA-IANBD), and staining of nuclei by propidium iodide (PI) or Toto-3 Iodide. **c** Exemplary single-cell traces typical for early markers (blue solid line) and late markers (red solid line) with the corresponding noise levels (dotted horizontal green lines). For early markers, the solid green line shows the initial slope, and the vertical black line depicts the breakdown time, which is the time from which on the trace deviates from the parabolic behavior (dashed green line) by more than a threshold. For late markers, the marker onset time (vertical black line) is the time when the onset tangent (diagonal solid green line) crosses the initial fluorescence level (horizontal solid green line)

Each fluorescence marker shows specific features in the intensity time traces. Figure 2a shows typical image time series for the six event markers monitored after exposure to 58 nm 100 µg mL^−1^ PS-NH_2_ nanoparticles. Despite the fact that each single-cell time trace is individual, all traces for a given marker exhibit common features, including characteristic saltations, which permit the determination of defined event times (Fig. 2b). We define the distinct onsets or sudden declines of fluorescence intensities as cellular “events” in the following. In order to extract the time points of these events (vertical black dotted lines) by means of an automated procedure, we use mathematical functions (gray dotted lines) that reproduce the featured fluorescent time traces including the abrupt onsets and precipitous drops in fluorescence intensities (Fig. 2c). We then apply a maximum likelihood estimation to extract best estimates of event times (Supplementary Fig. 1). Specifically, step functions multiplied with either algebraic or exponential functions were used as phenomenological model functions. For the early markers (LysoTracker, TMRM, and CellROX), the distinct declines in the signal (*t*_breakdown_) were determined by the time at which the curve deviated by more than a defined threshold. The late markers (Caspase 3/7, pSIVA-IANBD, and PI or Toto-3 Iodide) exhibited distinct onsets of the fluorescence, which were determined as the intersection points *t*_onset_ between the basal fluorescence and the tangent of the fit at half maximum. In the following, we describe the characteristic fluorescence time traces and the significance of the event sequences revealed by each marker.

The time traces of the first marker, LysoTracker, show an initial fluorescence increase described by a parabolic function, followed by a sigmoidal diminution of fluorescence. The marker is based on a pH-dependent fluorophore, which accumulates in cellular compartments such as lysosomes or late endosomes. In the acidic environment of the lysosomes, the acidotropic marker is protonated, which markedly enhances fluorescence. The breakdown of LysoTracker fluorescence indicates the time point of LMP, typically associated with lysosomal rupture, and is set as *t*_LMP_. The second marker, TMRM, shows a plateau of fluorescence followed by a breakdown similar to that seen for LysoTracker. The high initial fluorescence can be explained by the accumulation of TMRM in active mitochondria, which maintain an inner membrane potential Δ_Ψm_. The collapse of the fluorescence signal is correlated with the release of TMRM upon loss of the membrane potential. The time point of the sudden decline in fluorescence designated as *t*_MOMP_, which can be attributed to MOMP. The third marker of the initiation phase of programmed cell death, CellROX, reflects levels of oxidative stress. CellROX is weakly fluorescent in the reduced state and exhibits strong fluorescence upon oxidation in presence of oxygen radicals. Hence, the fluorescence signal is an indicator of the cytosolic and mitochondrial concentrations of ROS, and the initial rise in fluorescence corresponds to ROS production. The observed breakdown in CellROX fluorescence at later times is most likely related to the mitochondrial oxidative burst^28^, which leads to a massive release of ROS that destroy or quench the marker. We denote the time point of CellROX breakdown as *t*_OxBurst_. The next marker, CellEvent Caspase 3/7, is the first marker for the late phase in programmed cell death. The fluorescence shows a sharp sigmoidal onset, which is followed by a smooth decrease. Caspase 3/7 is a cell-permeable marker with a DEVD peptide sequence (asparagine-glutamine-valine-asparagine)-binding site. The peptide is cleaved upon activation of caspase 3/7, and following its diffusion into the nucleus, it shows strong fluorescence when intercalated into nuclear DNA. The exponential decay of fluorescence is probably caused by photo-bleaching. We denote the onset time by *t*_CASP-3_. Next, the fluorescence time traces of the Annexin-V-based pSIVA-IANBD marker are well described by the same sigmoidal increase as the caspase signal, but with a delay in signal decay. The marker fluoresces only when bound to PhS^29,30^. PhS is transposed from the inner to the outer leaflet of the plasma membrane bilayer during cell death as an “eat-me” signal for phagocytes^31^ (PhS-Flip). The marker for the final stage in cell death is PI or Toto-3 Iodide. Both compounds act by binding specifically to DNA and nonspecifically to RNA, which leads to the observed sharp increase in signal. The smooth descent of the PI or Toto-3 Iodide signal is due to unspecific staining and degradation of RNA^32^. The plasma membrane is impermeable to the chromophores in living cells, hence the PI or Toto-3 Iodide onset time indicates the time point of loss of plasma membrane integrity, *t*_PMP_^29^. The fluorescence in the cell, in parallel with the course of the fluorescence intensity, exemplary for the LysoTracker and the TMRM marker for A549 cells treated with 100 µg mL^−1^ PS-NH_2_ nanoparticles, can be seen in the Supplementary Movie 3.

### Distribution of cell death marker onset times

**Fig. 3:**
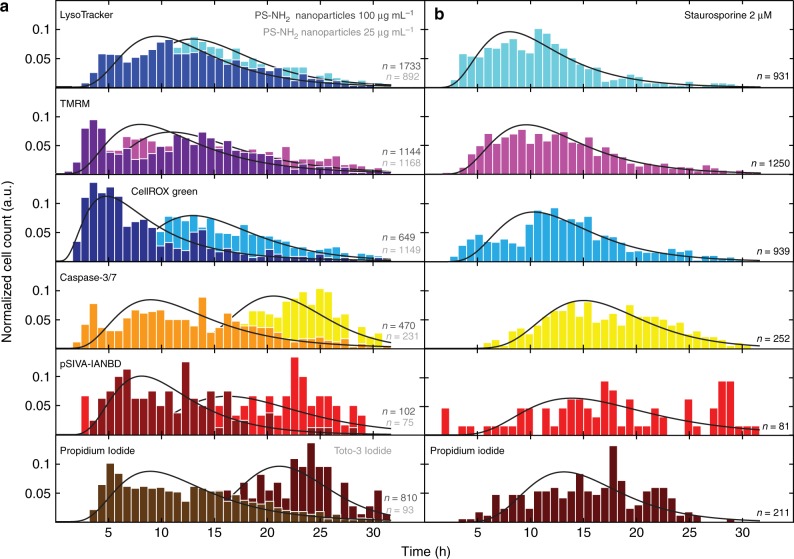
Distributions of the event times and their evolution over a period of 30 h. Normalized event time distributions for events visualized with the indicators LysoTracker, tetramethylrhodamine methyl ester (TMRM), CellROX green, Caspase 3/7, pSIVA-IANBD, and propidium Iodide or Toto-3 Iodide (nuclear DNA/RNA) after exposure to 25 and 100 µg mL^−1^ amino-modified polystyrene nanoparticles (PS-NH_2_ nanoparticles) (**a**) or 2 µM STS (**b**). Continuous lines indicate approximated log-normal distributions. Each individual plot shows pooled data from up to six individual experiments of cells, which showed both marker signals (*n*)

Time traces of the six cell death-related markers were analyzed for their respective event timing induced by exposure to PS-NH_2_ nanoparticles at a lower dose (25 µg mL^−1^) or a higher dose (100 µg mL^−1^). The distributions of event times are shown in Fig. 3a. For comparison the distribution in the case of exposure to STS (2 µM) is shown in Fig. 3b, as a control. Event times were determined as described above from fluorescence time series of more than 600 cells per early marker and in average 250 cells per late marker. The data for each marker originate from several measurements with different marker combinations and cells with both signals. The histograms are approximated by log-normal distributions, which are indicated in Fig. 3 (black line). Details on the log-normal distributions are given in the Supplementary Information. The histograms are arranged in the expected order of the corresponding events, such that early to late markers are shown from top to bottom. For cells exposed to 25 µg mL^−1^ nanoparticle, the maxima of the distributions appear between 11 and 13 h for the early markers and at around 16 and 21 h for the late markers. For cells treated with 100 µg mL^−1^ nanoparticles, the maxima of event times shift to earlier times and are found between 5 and 9 h for the early markers and at 8 and 9 h for the late markers. The dose-dependency of the timing is in agreement with our earlier findings^26^. Cell death induced by STS shows a temporal sequence of marker activation between 8 and 10 h for early markers and 13 and 15 h for late markers. See Supplementary Table 2 for the complete statistics of the event time distribution maxima. As fluorescent dyes and in particular the addition of dual markers are under suspicion to exert cellular stress and might affect the general cell viability, careful consideration was given to data statistics. In all experiments, negative controls without nanoparticle exposure have been carried out. The percentages of cells with both signals are low as given in Supplementary Table 3. We also noticed small but measurable effects of marker combinations on the event time distributions as shown in the Supplementary Figure 2. The distribution of a particular marker in some cases slightly shifts depending on the type of co-delivered second fluorescent marker. We also analyzed the cellular response timing with respect to day-to-day variance. The reader will find representative data for identical marker combinations measured on different days in Supplementary Figure 3. In general, careful considerations regarding the outcomes should be done already in the stadium of the experimental design, e.g. such as the type of nanoparticles, the choice of cell line, and the dose of nanoparticles added to the cells.

### Event pair correlations reveal order of sequence and timing

**Fig. 4:**
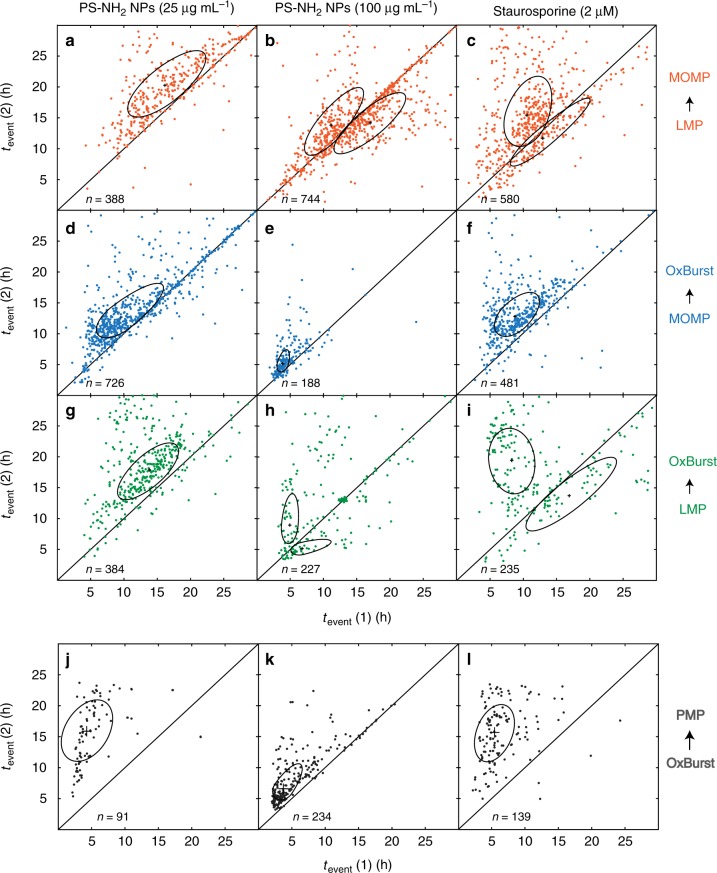
Pairwise fluorescence marker correlations of single-cell events in A549 cells. Two-dimensional representation of event times *t*_event_ (1) and *t*_event_ (2) of pairwise markers induced by exposure of cells to 25 µg mL^−1^ nanoparticles (left), 100 µg mL^−1^ nanoparticles (middle), or STS (right). The uniaxially asymmetric ellipses display a one-sigma interval around the cluster center. Mitochondrial outer membrane permeabilization (MOMP) was correlated with lysosomal plasma permeabilization (LMP) (**a**, **b**, **c**) as well as with oxidative burst (OxBurst) (**d**, **e**, **f**). Furthermore, LMP and OxBurst were correlated with each other (**g**, **h**, **i**). Cells treated with 25 µg mL^−1^ nanoparticles show weaker correlations between OxBurst and PMP (**j**), whereas a stronger correlation is observed for 100 µg mL^−1^ nanoparticles (**k**). A weak correlation is shown for staurosporine-exposed cells (**l**). *n* is the number of cells shown in the respective scatter plot

In Fig. 3, the relative shifts in the event time distribution maxima are small compared to the distribution widths. Moreover, in some cases the log-normal distributions do not approximate the true event time distributions sufficiently well. Hence, the average values of event times do not satisfactorily reflect the sequence of events. Instead, pairwise event time correlations between events (1) and (2) of two different pairs of markers are more likely to reveal the order of events. In Fig. 4, the parameter *t*_event_ (2) is plotted against *t*_event_ (1) in scatter plots arranged as a matrix whose rows represent different paired combinations of the events LMP, MOMP, OxBurst, and PMP, and whose columns represent the three conditions 25 µg mL^−1^ nanoparticles, 100 µg mL^−1^ nanoparticles, and (for comparison) 2 µM STS to which the A549 cells were exposed. The scatter plots are split into two halves by a diagonal line in order to indicate whether event (2) follows or precedes event (1); consequently, data points falling onto the diagonals represent temporal coincidence of the two events. The centers and widths of pairwise event distributions are visualized by ellipses derived from a principal component analysis (more details in Supplementary Fig. 4). The semi-axes of the ellipses are oriented along the two principal components. Their lengths indicate the mean squared displacement of events from the ellipse center in the direction of the corresponding semi-axis. In order to account for the asymmetric distributions of some clusters, we allowed the major semi-axis, which is oriented along the first principal component, to have a different length in positive and in negative direction, which results in asymmetric ellipses. Event clusters are identified by mean shift clustering (details are given in the Supplementary Information). Each scatter plot is based on data for mostly more than 100 cells, in average for more than 300 cells. Note that all data in Fig. 4 comes from A549 cells in which events were clearly detectable for both markers. Hence, cells that showed a non-evaluable signal due to low signal-to-noise ratio for at least one marker are not included. In some cases, the fluorescence signal was too low because the concentrations of the markers and of nanoparticles or STS were chosen to be very low in order to avoid non-specific effects of the experimental setup on cell behavior. Cells with undefined behavior, e.g. cells that showed partial detachment during cell death, were also omitted. The numbers of cells evaluated for each condition are listed in Supplementary Table 4.

The principal component analysis reflects the average behavior in event timing. If the center of the ellipse is located above the diagonal line, then the event plotted on the vertical axis (on average) follows the event plotted along the horizontal axis, and vice versa. Moreover, if the ellipse is elongated along the major semi-axis parallel to the diagonal, the event times are positively correlated, i.e. event 2 follows event 1 with a defined delay time. Hence, the pairwise event time correlations hint at a preferred sequence of events in A549 cell death. In the case of low (25 µg mL^−1^) nanoparticle dose this event cascade is given by LMP-MOMP-OxBurst (Fig. 4a, d, g). The measured average delay time between LMP-MOMP at the lower dose is 4 h (Fig. 4a), for MOMP-OxBurst it is 3 h (Fig. 4d), and the average time that elapses between LMP and OxBurst is about 4 h (Fig. 4g). The order of events and the defined timing are consistent with activation of the proposed lysosomal apoptotic pathway, suggesting that the cationic PS-NH_2_ nanoparticles trigger the rupture of lysosomes and subsequently induce MOMP. At higher nanoparticle dose (100 µg mL^−1^) the delay times of the lysosomal pathway shift to shorter intervals, i.e. 3 h for LMP-MOMP (Fig. 4b), 1.5 h for MOMP-OxBurst (Fig. 4e), and 4 h for LMP-OxBurst (Fig. 4h). Moreover, for the higher dose, a bimodal behavior emerges (Fig. 4b, h). A subset of cells below the diagonal can be discerned in which the order of events is reversed, i.e. MOMP (Fig. 4b) and Oxburst precede LMP (Fig. 4h). In the first case, LMP follows the MOMP with a delay of 3 h and LMP follows OxBurst by 2 h. As a control, we compare these data to those for STS-induced cell death (Fig. 4c, f, i), which are similar to the results for nanoparticles at the higher dose. For example, here is also a bimodal behavior detected for the correlation between LMP and MOMP (Fig. 4c). Around one-half of the cells showed the sequence MOMP-LMP (Δ*t* = 1.5 h), whereas the other half displayed MOMP following LMP with a delay time of 5 h. The duration of the delay between MOMP and OxBurst is 4 h (Fig. 4f). Moreover, for the combination LMP-OxBurst we see also a distinct partition into two clusters (Fig. 4i). The first cluster LMP-OxBurst has a delay time of 11 h, while the second cluster OxBurst-LMP is delayed by 3 h.

We conclude that the nanoparticle exposure with a lower concentration (25 µg mL^−1^) triggers in A549 cells an unidirectional and highly correlated LMP-MOMP-OxBurst event sequence in agreement with the notion of a lysosomal apoptotic pathway. At higher (100 µg mL^−1^) nanoparticle exposure, the LMP-MOMP-OxBurst response also prevails but is shifted to earlier times and in addition, a second event cascade with opposite order MOMP-OxBurst-LMP appears. The dominant LMP-MOMP-OxBurst lysosomal pathway is also present in experiments using a second cell line, the Huh7 human hepatocarcinoma cell line, for comparison (Supplementary Figure 5). For the lower concentration (25 µg mL^−1^) we found the same order of events for Huh7, but slightly longer delay times than for A549 cells. Huh7 cells also exhibit a shift of the LMP cascade to earlier times at higher (100 µg mL^−1^) nanoparticle exposure. Interestingly, however, there is no evidence of bimodal behavior, since the MOMP-OxBurst-LMP sequence is missing, indicating that under this conditions in Huh7 cells the mitochondrial signaling cascade is not triggered.

Next, we are interested in the correlations of the event times with the final event of cell death. For the model cell line A549 Fig. 4j shows that the correlation of the early event (OxBurst) with the late event PMP is weaker correlated at lower dose and stronger correlated at the higher nanoparticle concentration (Fig. 4k). For STS (Fig. 4l) also a weaker correlation is observed. The delay times for the OxBurst-PMP correlations are 12, 3, and 10 h for 25 µg mL^−1^ nanoparticles, 100 µg mL^−1^ nanoparticles, and STS, respectively. In contrast to the early markers, the three later markers (Caspase 3/7, pSIVA-IANBD, and PI or Toto-3 Iodide) show strong short-timed correlations (Supplementary Fig. 6). However, as soon as a late marker is correlated with an early marker, the scattering is again observed (Supplementary Fig. 7). This fact indicates that much of the noise that leads to heterogeneous timing is generated during the initiation phase. A complete overview of the positions of all ellipse centers of Fig. 4 is given in Supplementary Table 5, the mean shift clustering properties in Supplementary Table 6. The complete statistics concerning the Huh7 cells can be found in Supplementary Table 7. In the Supplementary Tables 8-12 mean shift properties, statistics, and delay times of Supplementary Figures 6 and 7 are shown.

### Production of ROS is correlated with MOMP timing

**Fig. 5:**
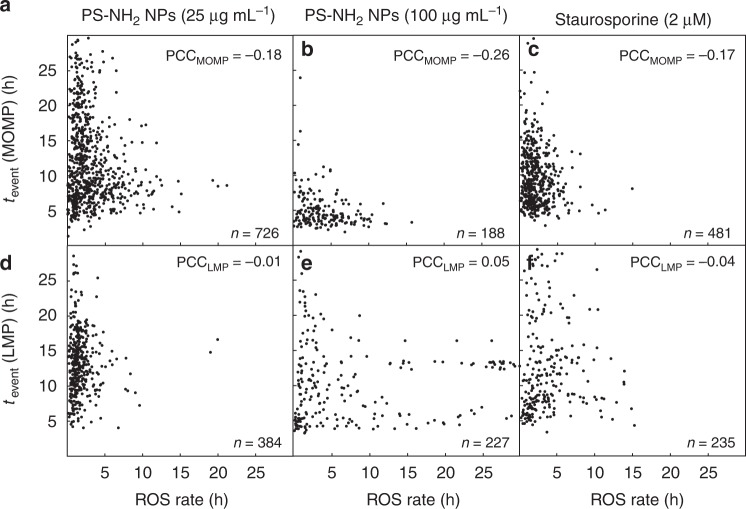
Rates of reactive oxygen species (ROS) production correlate with mitochondrial outer membrane permeabilization (MOMP), but not with lysosomal membrane permeabilization (LMP). A549 cells were treated with 58 nm amino-modified polystyrene nanoparticles (PS-NH_2_ nanoparticles) at 25 µg mL^−1^ (**a**, **d**) or 100 µg mL^−1^ (**b**, **e**), or with STS (**c**, **f**). The ROS production rates were determined from the linear increase in the CellROX fluorescence and plotted versus the time of outer mitochondrial membrane breakdown (*t*_event_(MOMP)) or lysosomal leakage (*t*_event_(LMP)). An inverse relationship between ROS production rate and MOMP was observed for cells treated with nanoparticles at 25 and 100 µg mL^−1^ (**a**, **b**) as well as for STS (**c**). In contrast, no correlation was observed between ROS production rate and LMP (**d**–**f**). In the upper right corner, the Pearson correlation coefficient (PCC) is depicted for each plot, respectively. *n* is the number of cells shown in the respective plot

ROS play a crucial role in apoptosis, as they are generated in many compartments of the cell. Under physiological conditions, only a low level of ROS, which is necessary i.e. for messenger signaling, is present in the cell. A high ROS level, however, leads to cell death^33^. In particular, accumulation of ROS is believed to lead to enhanced permeabilization of lysosomes and cause damage to mitochondria^20,34^. Since the initial slope of fluorescence time traces for the CellROX marker is assumed to reflect the rate of increase of ROS in single cells, we examined whether the rate of ROS production correlates with the onset time of MOMP or LMP in A549 cells (Fig. 5). The slope of the line through the first two points returned by the CellROX model function, fitted to the time trace and evaluated with high temporal resolution (1 min) (Supplementary Fig. 1) was taken as a measure for the ROS production rate. In Fig. 5a–c, the event time of MOMP is plotted against the ROS production rate for the conditions 25 µg mL^−1^ nanoparticles, 100 µg mL^−1^ nanoparticles, and STS. In all three conditions, we see that cells with elevated ROS production tend to show MOMP at earlier time points (Fig. 5a–c). The Pearson correlation coefficients (PCCs) are −0.18, −0.26, and −0.17, respectively. Details on the PCC are given in the Supplementary Information. The remarkable negative correlations could be explained if we assume that a critical threshold of the ROS level leads to MOMP. As a result, at higher ROS production rate the maximum ROS level is reached at an earlier time and consequently an earlier MOMP event time. In contrast, the LMP events are essentially independent of the ROS production rate, which is indicated by very low PCC values (Fig. 5c–f).

### Higher PS-NH_2_ nanoparticle levels trigger lysosomal and mitochondrial pathways

**Fig. 6:**
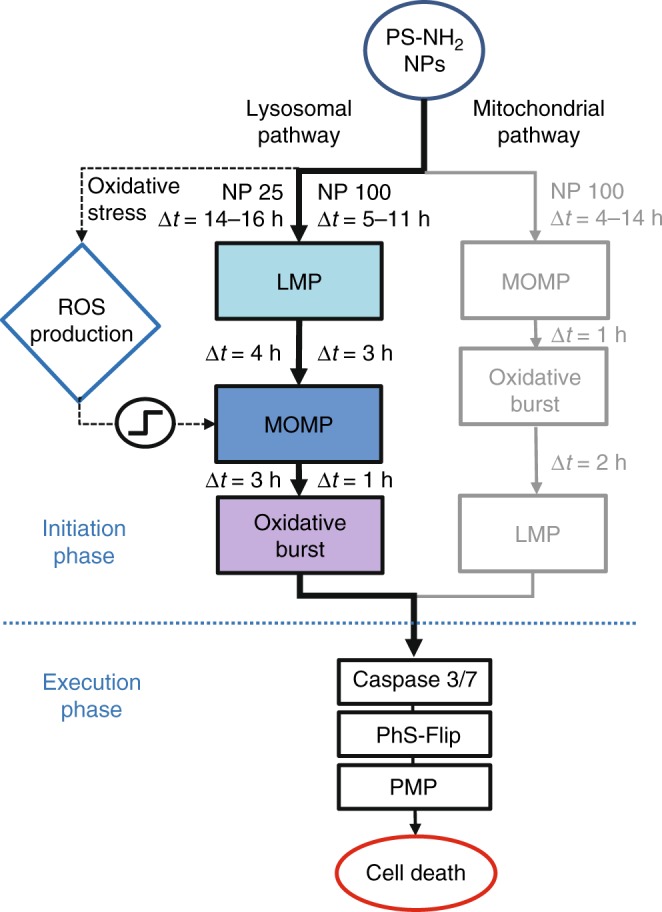
Proposed model for the relationships between lysosomal membrane permeabilization (LMP)-dependent and mitochondrial outer membrane permeabilization (MOMP)-dependent cell death pathways in A549 cells exposed to the lower and the higher doses of 58 nm amino-modified polystyrene nanoparticles (PS-NH_2_ nanoparticles). In the first case (25 µg mL^−1^), the internalization of PS-NH_2_ nanoparticles results in reactive oxygen species (ROS) production and accumulation of nanoparticles in lysosomes, which activates the LMP-dependent cell death pathway. Both LMP and ROS production can contribute to the induction of MOMP. As the last step in the initiation of the LMP-dependent pathway, the release of mitochondrial contents initiates the oxidative burst, which cause ultimately cell death. The application of the higher dose of nanoparticles (100 µg mL^−1^), in addition to the abovementioned process, the MOMP-dependent pathway can be triggered directly, i.e. independently of LMP and ROS, thus inducing an oxidative burst which is followed by LMP. The LMP- and MOMP-dependent pathways converge in the execution phase to activate caspase 3/7 and the phosphatidylserine flip to the outer membrane, and finally lead to cell death

We focused on the measurement of event time differences of pairwise combinations of multiple fluorescent cell death markers. In Fig. 6, we show a schematic pathway diagram that summarizes the observed delay times and proposes a hypothetical order of events measured in A549 cells. The dominant signaling pathway is the lysosomal pathway (LMP-MOMP-OxBurst), which is the only pathway induced at the lower nanoparticle dose (25 µg mL^−1^). In contrast, at higher nanoparticle dose (100 µg mL^−1^) and for STS, not only the lysosomal pathway but also a mitochondrial pathway (MOMP-OxBurst-LMP) comes into play. These findings are in general agreement with previous reports on PS-NH_2_ nanoparticles for a comparable dose and cell line^3,4^. The LMP-MOMP-OxBurst sequence, as well as the timing of events, is consistent with the general belief that a lysosomal apoptotic pathway proceeds through mitochondria^17^. Amino-modified nanoparticles provoke swelling of the lysosomes, followed by lysosomal collapse and most probably the release of lysosomal proteases like cathepsin-D^9^. It is known that destabilization of lysosomal membrane mediates apoptosis induced via mitochondria with consequent production of ROS^35^. However, it has been suggested that nanoparticles also damage mitochondria directly^16^. Our higher-dose data support the existence of this second pathway. Likewise for STS-induced apoptosis, both pathways have been implicated and it is known that cytosolic cathepsin-D is a key mediator^36^. Single-cell event correlations confirm that both pathways are involved and in principle allow for quantification of their respective contributions.

The role of intracellular ROS in nanoparticle-induced cell death is still under debate. Exposure to nanoparticles is known to increase levels of ROS, which are recognized to sensitize the cell death mechanism. It has been shown that the LMP pathway has a feedback loop for ROS, and that ROS is increased by the effects of LMP/cathepsin-D release on mitochondria^37^. Clearly, the formation of ROS depends on nanoparticle dose and exposure time^28^. The finding that ROS production is negatively correlated with the MOMP event times in A549 cells supports the notion that a critical level of ROS activates MOMP (Fig. 6, on the left). Hence, ROS damage feeds into the mitochondrial node, confirming the role of MOMP as central integrator of the cell death pathway^11–13,15^. We attributed the delayed abrupt drop of the ROS marker signal to mitochondrial rupture, which is understood to play an important role in the downstream signaling pathways^38^. Hence, mitochondria are both sources and targets of ROS, as previously noted^39^. The measured relation between ROS production and LMP event time does not substantiate the hypothesis that ROS destabilizes the lysosomal membrane. In light of the substantial noise seen in the correlations during the initiation phase, in both nanoparticle-induced as well as STS-induced cell death, the existence of other, extrinsic or intrinsic factors must be assumed that affect the susceptibility of cells.

We stress that the temporal correlation of events does not necessarily imply a causal relationship between events. Nevertheless, correlations help to evaluate hypothetical models, which can be discarded if the correlations that should follow predicted causal relationships are absent or appear contradictory. As an example, we refer to the observed coexistence of two clusters on opposite half-spaces in the event time correlation plane. Since in a causal relationship, event A cannot precede event B and at the same time B precede A, we must assume two independent mechanisms with opposite temporal order. Another lesson learnt from the observed broad spread in event time correlations is that the pathways are sequences of stochastic processes. When measured at the single-cell level, the variance in early cell death responses is striking, and the presence of cross talk and hidden determinants results in broadly scattered cell death times. As such, the analysis of single-cell event timing reveals previously inaccessible short-term correlations, such as defined delay times between two sequential steps and hence, we believe, it should as well be applicable in other biological fields. It is interesting to note that single-cell time-lapse microscopy in principle can even resolve much faster event cascades down to millisecond resolution.

## Discussion

Single-cell analysis of event time correlations proofs to be a versatile tool that provides the statistics of event order and timing in signaling pathways. In the case of nanoparticle-induced cell death discussed here, the ellipsoidal clusters in the two-dimensional event representations confirm that clearly a sequentially ordered LMP-MOMP-OxBurst lysosomal pathway exists at 25 and 100 µg mL^−1^ nanoparticle exposure (58 nm) in A549 cells. At higher nanoparticle exposure, event times are shifted to earlier times meaning that higher dose accelerates the lysosomal damage. More importantly at a higher dose in the case of A549 cells, event clusters appear in the opposite half-spaces of the two-dimensional representation points. These clusters hint at the coexistence of a second, mitochondrial pathway by the same stimulus, which could not be disentangled previously. Our results suggest that a higher dose of 58 nm PS-NH_2_ nanoparticles induces A549 cell death via the lysosomal pathway as well as the mitochondrial pathway. Interestingly, the second mitochondrial pathway was not evident in Huh7 cells, but the lysosomal signaling cascade, valid for both nanoparticle concentrations. Data on the timing of cell death-related events is expected to inform not only toxicology but also anticancer or immunology drug development, and might help to resolve issues relating to drug resistance in subpopulations. The role of the timeline in the impact of drugs, and its implications for drug combinations that target specific organelles at defined time points remains poorly explored. Single-cell time correlations yield a dynamic fingerprint of signal progression, which is capable of tracing the impact of nanomaterials on mechanistic pathways and elucidating their interdependencies with higher precision. As such, the method promises to provide a considerable contribution to a systems-based understanding of signal transduction cascades.

## Methods

### Cell culture

The A549 cell line (American Type Culture Collection) was cultured in modified Eagle’s medium with Earle’s salts and 2 mM l-glutamine, and Huh7 cell line (I.A.Z Toni Lindl GmbH) was cultured in Roswell Park Memorial Institute 1640 Medium with GlutaMAX^TM^ Supplement, 5 mM HEPES, and 1 mM sodium pyruvate with 10% fetal calf serum (FCS) respectively. Both cell lines were tested negative for mycoplasma contamination, but not authenticated. Cells were grown to 70–80% confluence, trypsinized with 0.5% trypsin/0.2% EDTA or Accutase solution and centrifuged at 1000 rcf for 3 min. Cell pellets were resuspended in cell medium with 10% FCS and seeded in the microstructured slides.

### Fluorescent markers

Lysosomes were stained with LysoTracker Deep Red (75 nM, ThermoFisher), the mitochondrial membrane potential was visualized with TMRM (10 nM, Sigma-Aldrich), the activation of caspase 3/7 detected with CellEvent Caspase 3/7 (3%, ThermoFisher), the OxBurst/ROS level with CellROX green (100 nM, ThermoFisher), and the flipping of PhS was monitored with the pSIVA-IANBD indicator in the Polarity Sensitive Indicator for Viability and Apoptosis Kit (2%, Novus Biologicals). The impermeant dye PI (1%, Novus Biologicals) or Toto-3 Iodide (1 µM, Life Technologies) was used to visualize nuclei. In some cases, the nuclei of cells were stained with 25 nM Hoechst 33342 (Life Technologies). All dyes were applied without any further washing step.

### Physico-chemical characterization of PS-NH_2_ nanoparticles

The PS-NH_2_ nanoparticles with a diameter of 58 nm, dispersed in deionized water, were used as a model for cationic nanoparticles to induce cell death and were purchased from Bangs Laboratory, USA. The nanoparticle dispersion was stored at 4 °C and used as provided without any additional purification. The same batch of nanoparticles was used for all experiments. The physico-chemical characterization of such particles (same material, coating and solvent and similar size) was published elsewhere before^3–7,10,40,41^. In summary, the (hydrodynamic) size was characterized by dynamic light scattering as well as with transmission electron microscopy (TEM)^6^. In both measurements, the nanoparticles were well dispersed in phosphate-buffered saline, indicated by the TEM images and by a low polydispersity index. Ruenraroengsak et al. found that the PS-NH_2_ nanoparticles are also monodispersed with just small aggregates in distilled water as well as in tissue culture medium^5^. Furthermore, it was shown that the addition of FCS does not affect the monodispersity of nanoparticle solutions^10^. Moreover, the cationic nanoparticles showed a positive charge over a broad pH spectrum in aqueous solution, analyzed by ζ-potential measurements^6^. The nanoparticles were administered to the cells in Leibovitz’s L15 medium supplemented with 10% FCS, to keep the experiment conditions as close as possible to the cell culture conditions, and to allow the formation of protein corona on the surface of the nanoparticles.

### Reagents

STS (2 µM), used as a positive control for cell death, was purchased from Sigma-Aldrich.

### Single-cell microstructured slides

Microstructured arrays were produced by µPIP following the protocol published in Röttgermann et al.^27^. Briefly, polydimethylsiloxane stamps were placed in eight-well slides (ibidi GmbH) as a mask and selectively treated with oxygen plasma (Femto Diener, 35–50 W for 3 min).The plasma-exposed areas were then passivated for 25 min with (2 mg mL^−1^) PLL(20k)–*g*(3.5)-PEG(2k) (SuSoS AG) in aqueous buffer (10 mM HEPES (pH 7.4) and 150 mM NaCl). All other areas were made cell-adherent by exposure to 35 µg mL^−1^ (A549) or 50 µg mL^−1^ (Huh7) fibronectin (YoProteins) for 45 min. After incubation with cell culture media, wells were seeded with 8000 cells each, equivalent to a filling of roughly 1 cell per adhesion site. Non-adherent cells were washed off with fresh medium for optimal occupancy. Cells were incubated at 37 °C for 6 h before drug and marker administration. The geometry of the square adhesion sites was 30 × 30 µm and the lattices were placed 90 µm apart.

### Automated image acquisition

Time-lapse image series were recorded using an inverted TI Eclipse microscope (Nikon) to scan around 150 positions (fields of view at ×10 magnification) per sample over a period of 30 h. For each position, a phase-contrast image and a maximum of three fluorescent images were acquired every 10 min by a ClaraE charge-coupled devise camera. The cell culture slide was maintained at 37 °C by an ibidi heating system.

### Image and data analysis

NIS Elements AR 5.02.00 64-bit was used to obtain time-lapse microscopy measurements and to convert nd-files (proprietary Nikon format), using a custom macro, into TIFF format. To extract single-cell fluorescence time traces from the time-lapse image series, a region of interest (ROI) grid corresponding to the adhesion sites was adjusted to the images using a customized plugin for ImageJ^42^. Then, each ROI occupied by exactly one cell was marked for evaluation in a semi-automated manner. This quality-control step helps to avoid the evaluation of ROIs occupied by no, by multiple, or by displaced cells, and ensures appropriate single-cell readout. Then, a background correction was applied to the image series, and the time trace of the total integrated fluorescence intensity was determined for each ROI marked for evaluation. Using maximum likelihood estimation, marker-dependent model functions were fitted to the time traces in order to retrieve the event times of the fluorescent markers (Supplementary Fig. 1). The model functions describe the fluorescence time courses of the markers using a combination of a sigmoidal function with a parabola or an exponential function. The best fits are then analyzed to extract the event times or to discard time traces for which no events could be found. Details on the event time extraction are given in the Supplementary Information. The time delay between drug administration and the first measurement was added to the event times. Clusters of the pairwise correlated event times were identified by mean shift clustering^43^. The cluster centers were visualized by ellipses with asymmetric major semi-axes in order to account for anisotropic scattering of the data. Details of the determination of clusters and ellipses can be found in the Supplementary Information.

### Code availability

The in-house written code for the fitting software is available under the following 10.5281/zenodo.1418465. In addition, the plotting software is accessible with this 10.5281/zenodo.1418463.

## Supplementary Information

Supplementary Information

Description of Additional Supplementary Files

Supplementary Movie 1

Supplementary Movie 2

Supplementary Movie 3

## Data Availability

The datasets generated and/or analyzed during the current study are available in the Zenodo repository, namely with this 10.5281/zenodo.1418377. Raw image data will be made available upon request.
