# Supplementary material for: A high-throughput microscopy method for single-cell analysis of event-time correlations in nanoparticle-induced cell death
Source: Commun Biol. 2019 Jan 24;2:35. doi: 10.1038/s42003-019-0282-0 (PMC6345847; doi:10.1038/s42003-019-0282-0)
Supplement: Supplementary file 1 — Supplementary Information [file 42003_2019_282_MOESM1_ESM.pdf]

Contents:

Supplementary Figure 1: Mathematical procedure for determination of event-times.

Supplementary Figure 2: Calculation of ellipses of concentration.

Supplementary Figure 3: Influence of marker combinations for early markers.

Supplementary Figure 4: Reproducibility of data.

Supplementary Figure 5: Pairwise fluorescence marker correlations of single Huh7 cells.

Supplementary Figure 6: Correlation of PMP with PhS-Flip and CASP-3.

Supplementary Figure 7: Correlation of late markers.

Supplementary Table 1: Low concentration of PS-NH_2 of 10 µg mL^{-1}.

Supplementary Table 2: Overview of maxima of event-time distributions shown in Figure 3.

Supplementary Table 3: Statistics of negative controls.

Supplementary Table 4: Overview of statistics of Figure 4.

Supplementary Table 5: Delay times of scatter plots shown in Figure 4.

Supplementary Table 6: Mean shift clustering properties used for Figure 4.

Supplementary Table 7: Complete overview of Huh7 data (Supplementary Fig. 5).

Supplementary Table 8: Mean shift clustering properties of Supplementary Figures 6 and 7.

Supplementary Table 9: Statistics of Supplementary Figure 6.

Supplementary Table 10: Delay times of scatter plots shown in Supplementary Figure 6.

Supplementary Table 11: Statistics of Supplementary Figure 7.

Supplementary Table 12: Delay times of scatter plots shown in Supplementary Figure 7.

Mathematical functions used for phenomenological fits of experimental time traces

In the main text, the characteristic signatures of the single-cell traces are discussed in terms of the occurrence of distinct onsets and drops in the fluorescence signal. The corresponding event times were not extracted directly from the traces, but from best fits to marker-specific phenomenological functions. In this section, we describe the mathematical functions used to fit the traces and the rules we used to exclude outliers.

The six markers are divided into two classes, based on their characteristic behaviour: the early markers (LysoTracker, TMRM, and ROS) and the late markers (Caspase 3/7, pSIVA-IANBD, and PI/Toto-3 iodide). Representative time traces for the markers, together with the corresponding fits are shown in **Supplementary Figure 1**.

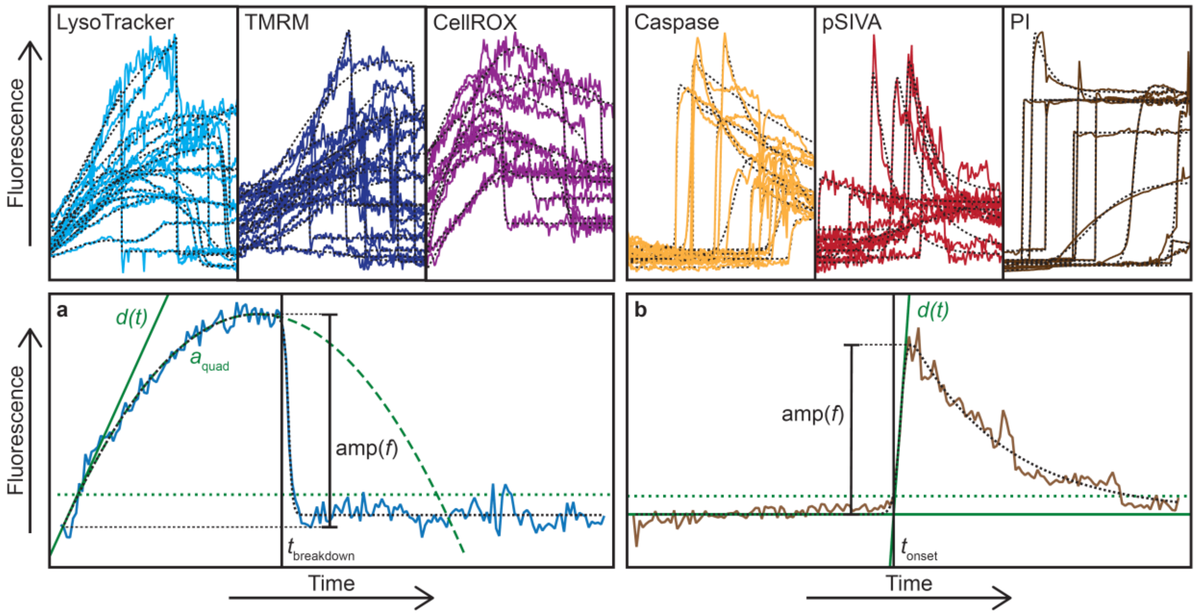

Supplementary Figure 1: Mathematical procedure for determination of event times. Representative time traces for the various markers (LysoTracker, TMRM, CellROX, Caspase 3/7, pSIVA-IANBD, and PI/Toto-3 iodide) showing the experimental data (colored lines) and the corresponding best fits (black dashed lines). The early markers are characterized by a parabolic ascent, followed by a steep decline (**a**), and the late markers by a steep onset followed by a slow decay (**b**).

Marker-specific functions were fitted by maximum-likelihood estimation assuming a Gaussian probability distribution[1] using Matlab[2].

To the traces of the early markers, the function

$$f(t) = \sigma(t) \cdot \left(a_{\mathrm{const}} + s\, a_{\mathrm{quad}}(t - t_{\mathrm{vertex}})^2\right) + \left(1 - \sigma(t)\right) \cdot b$$

was fitted, where

$$\sigma(t) = \frac{1}{1 + e^{\alpha(t_{\mathrm{step}} - t)}}$$

is a sigmoid function and a_{const}, s, a_{quad}, t_{vertex}, t_{step}, α and b are fit parameters. $\sigma(t)$ describes the transition from the parabola to the final value b with the sharpness $\alpha < 0$. The first term describes a parabola with constant coefficient a_{const}, quadratic coefficient a_{quad}, and its vertex at t_{vertex}. $s \in \{-1, +1\}$ determines the opening direction of the parabola and is fixed to $s = -1$ for LysoTracker and CellROX.

To the traces of the late markers, we fitted the function

$$f(t) = A + B \cdot \sigma(t) \cdot \left(e^{\gamma(t_{\mathrm{step}} - t)} + b\right),$$

with the fit parameters A, B, γ, t_{step}, b, and α.

The sigmoid function $\sigma(t)$ describing the rise of the fluorescence at time t_{step} with a steepness $\alpha > 0$ is the same as for the early markers. A is the global offset of the function, and B a scaling factor specifying the peak height. The signal decay caused by photobleaching is described by time t_{step}, a decay constant γ and an offset b between the initial level A and the final level.

For each fitted trace, the minimum value $\min(f) \coloneqq \min_{i=1,\dots,N}(f_i)$ and the maximum value $\max(f) \coloneqq \max_{i=1,\dots,N}(f_i)$ of the datapoints $f_i \coloneqq f(t_i)$ at the timepoints t_i for $i = 1, \dots, N$ are calculated. This allows one to define the amplitude of a trace as

$$\mathrm{amp}(f) \coloneqq \max(f) - \min(f).$$

The time corresponding to the maximum is denoted as $t_{\max}$.Moreover, we define the value at half amplitude $f_{1/2}$ as

$$f_{1/2} \coloneqq \frac{\min(f) + \max(f)}{2}$$

and the time $t^{<}_{1/2}$ at half amplitude prior to the maximum as

$$t^{<}_{1/2} \coloneqq t_i \,|\, i \in \{1, \dots, N\} \text{ such that } \left|f_i - f_{1/2}\right| \text{ minimal and } t_i < t_{\mathrm{max}}.$$

Definition of event times

Event times are the time points when onset or descent of the signal begins. An overview of the event times extracted from the fits is given in **Supplementary Figure 1**. In order to determine event times in a robust and automated way, a mathematical construct is required that allows one to calculate the time points from the best fits.

The fluorescence breakdown time $t_{\mathrm{breakdown}}$ of the early marker signals is defined as the time at which the deviation from the initial parabolic behaviour exceeds an empirical threshold Δp_{max}; the value chosen was 3% of the trace amplitude.

Let the parabola values $\{p_i\}_{i \in \{1,\dots,N\}}$ be given by

$$p_i = a_{\mathrm{const}} + s\, a_{\mathrm{quad}}(t_i - t_{\mathrm{vertex}})^2,$$

with the parameter values obtained from the fit. Then, the breakdown time is

$$t_{\mathrm{breakdown}} = \min_{i \in \{2,\dots,N-1\}} (t_i | p_i - f_i > \Delta p_{\mathrm{max}}).$$

However, if the value of α is below a threshold, this formula no longer yields reliable values. Therefore, if α is below a threshold, $t_{\mathrm{breakdown}}$ is defined as the time at which the steepest tangent to the falling edge reaches $\max(f)$ and is calculated by

$$t_{\mathrm{breakdown}} = \frac{\max(f) - f(t_{\mathrm{fall}})}{\left.\frac{\mathrm{d}f}{\mathrm{d}t}\right|_{t=t_{\mathrm{fall}}}} + t_{\mathrm{fall}},$$

where

$$t_{\mathrm{fall}} \coloneqq t_i \,|\, i \in \{1, \dots, N\} \text{ such that } \left.\frac{\mathrm{d}f}{\mathrm{d}t}\right|_{t=t_i} \text{ minimal and } t_i > t_{\mathrm{max}}.$$

The fluorescence onset time t_{on} of the late markers is the intersection of the ascent tangent at half maximum with the initial value, and is given by

$$t_{\mathrm{on}} := \frac{\left(f_1 - f_{1/2}\right)}{\left.\frac{\mathrm{d}f}{\mathrm{d}t}\right|_{t=t^{<}_{1/2}}} + t^{<}_{1/2},$$

where

$$\left.\frac{\mathrm{d}f}{\mathrm{d}t}\right|_{t=t^{<}_{1/2}} := \frac{f_{j+1} - f_{j-1}}{t_{j+1} - t_{j-1}},$$

with $j \in \{2, \dots, N-1\} \mid t_j = t^{<}_{1/2}$.

In addition to the event-times, also the trace amplitude and the event slopes were extracted from the fits, where "onset slope" is defined as the slope of the steepest tangent to the rising edge, and the "breakdown slope" is the slope of the steepest tangent to the falling edge.

Filter rules

In order to discriminate between traces that exhibit an event signal and traces that do not show a clearly identifiable event signal, filter rules were established. Any traces with amplitudes $\mathrm{amp}(f)$ larger than 100 times the median of amplitudes are discarded to eliminate outliers, as are traces with amplitudes smaller than 10% of the largest amplitude of the remaining traces, which can hardly be distinguished from noise. Another criterion relates to traces with transient, narrow peaks, which are caused by fluorescent particles floating through the image. In addition, traces that merely show a monotonously increasing or decreasing signal are filtered out, since they bear too little information to extract an event time. In addition, there are specific filter rules for the two marker classes. Early marker traces are discarded if the maximum value was attained at the first or at the very last time point of the measurement, if the steepness α is less than an empirical threshold, or if the fluorescence breakdown is too shallow. Late marker traces are discarded if the maximum value is reached before the minimum value, or if the fluorescence is changing too fast at the beginning or still increasing at the end of the measurement.

Histogram distributions

The histograms in **Figure 3**, **Supplementary Figure 3** and **Supplementary Figure 4** contain graphs of log-normal distributions fitting the event time distributions. Here, we describe how the properties of the distributions are obtained.

The probability density function $p(t|\mu,\sigma)$ of the log-normal distribution is

$$p(t|\mu,\sigma) = \frac{1}{\sqrt{2\pi}\sigma t} \exp\left(-\frac{(\ln(t)-\mu)^2}{2\sigma^2}\right)$$

with the distribution parameters σ and μ.

The distribution parameters are calculated from the n event time values $t_i, i = 1, \ldots, n,$ shown in the histogram, using the empirical expectation value E and the empirical variance V:

$$E = \frac{1}{n}\sum_{i=1}^{n} t_i\,, \quad V = \frac{1}{n-1}\sum_{i=1}^{n} |t_i - E|^2$$

The distribution parameters and the distribution maximum $\hat{t}_{max}$ are then given as:

$$\sigma = \sqrt{\ln\left(\frac{V}{E^2}+1\right)}, \quad \mu = \ln(E) - \frac{\sigma^2}{2}, \quad \hat{t}_{max} = \exp(\mu - \sigma^2)$$

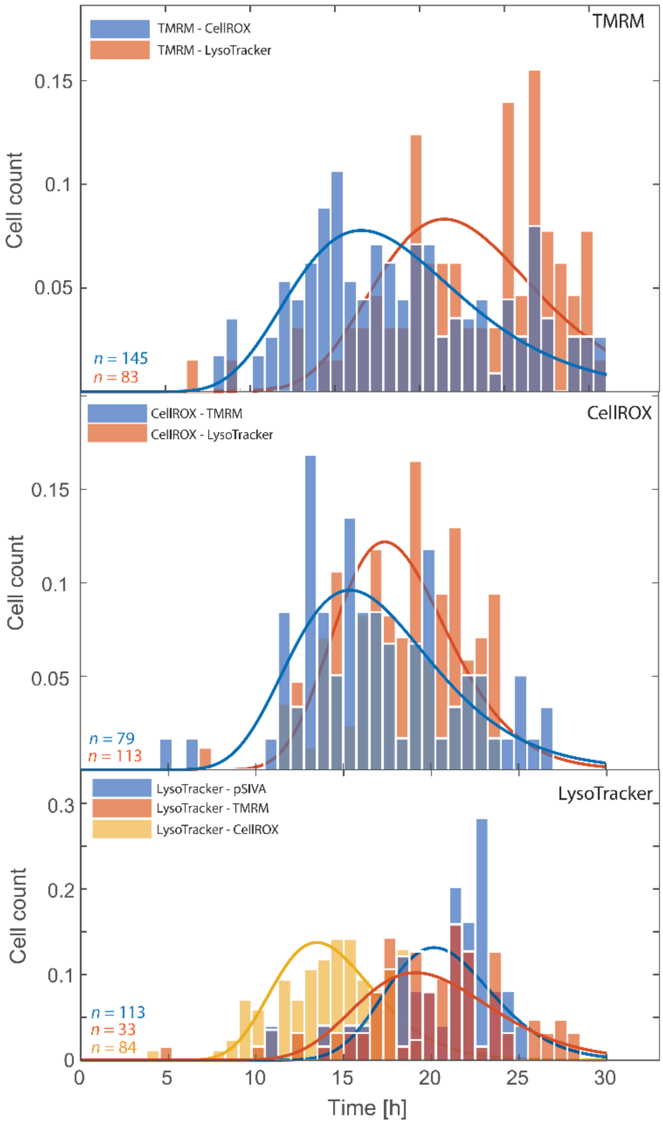

Supplementary Figure 2: Influence of marker combinations for early markers. The timing of events is plotted as a function of marker combinations. The event-time distribution of a given marker varies slightly with the marker combination. The different combinations of the particular markers were measured on the same day to exclude day-to day-variance.

The data shown here are from experiments on cells treated with the lower nanoparticle dose (25 µg mL^{-1}).

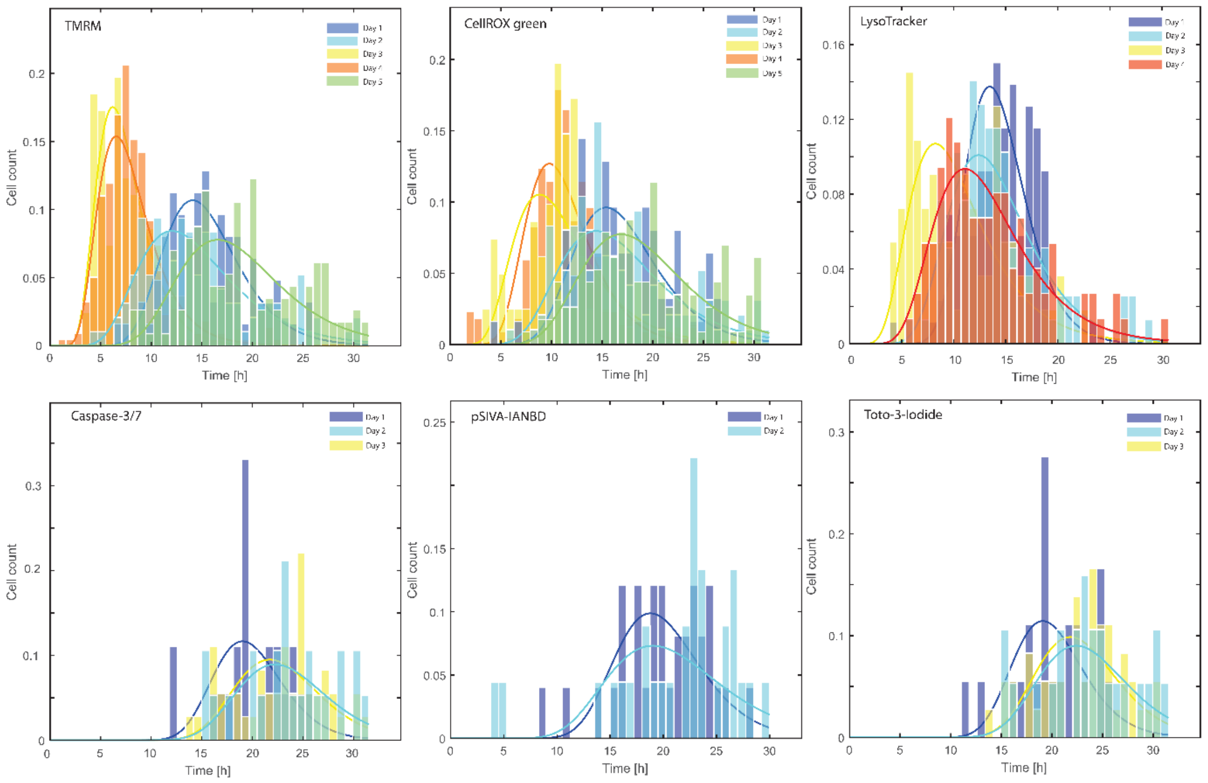

Supplementary Figure 3: Reproducibility of data. The event-time distributions of the used markers in the same combination respectively, measured on different days are shown. A549 cells were treated with 25 µg mL^{-1} PS-NH_2 nanoparticles together with one marker combination. For each marker, the distributions of the data from the single experiments (n = 3-5, pSIVA combination did not show double positive events in the third experiment) are plotted. The data show reproducibility of the general features within day-to-day variability. For TMRM and CellROX as well as for CASP and Toto-3 Iodide the combinations TMRM–CellROX and CASP–Toto-3 are shown, respectively. The distributions of the LysoTracker events stem from the LysoTracker–CellROX combination and the pSIVA events are derived from the pSIVA–LysoTracker combination.

Mean shift clustering of events

The event clusters in **Figure 4,** as well as in **Supplementary Figures 5, 6 and 7,** were identified by the mean shift algorithm[3]. The mean shift algorithm was performed in two dimensions using an Epanechnikov kernel. The bandwidths were chosen manually per dataset and are listed in **Supplementary Tables 6, 7 and 8**. The mean shift algorithm was applied to all points of a dataset until the mean shift was less than 0.02 h for each point. Then the clusters were identified so that all points separated from each other by less than a minimum distance of 10 minutes belong to the same cluster, and points in different clusters are separated by at least this minimum distance. When more than one cluster was found, only the largest cluster was used; the smaller clusters were considered as noise. Where indicated in **Supplementary Tables 6 and 8**, mean shift clustering was performed separately for the points below and above the diagonal, with points exactly on the diagonal being counted as below the diagonal, to prevent clusters from extending across the diagonal.

Ellipses of concentration

A principal-component analysis was carried out on each cluster in the two-dimensional event time plots to determine the average and variance of events. The ellipses of concentration are oriented along the eigenvectors of the covariance matrix and centered at the median of the corresponding cluster. The lengths σ of the semi-axes are the mean squared displacement of the n points $p_1,\ldots,p_n$ from the cluster median m in the direction of the corresponding semi-axis:

$$\sigma = \frac{1}{n}\sum_{i=1}^{n}(p_i - m)^2$$

For asymmetric semi-axes, only the points within the corresponding direction of the semi-axis are considered, and the length of the semi-axis is changed where the projection of the position vector on the asymmetric semi-axis disappears (**Supplementary Fig. 2**).

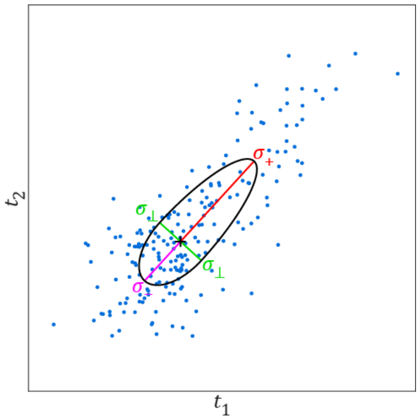

Supplementary Figure 4: Calculation of ellipses of concentration. An asymmetric ellipse of concentration (black) in a cluster of random points (t_1,t_2) is shown. The median of the points is indicated by the black cross. The length $\sigma_\perp$ of the symmetric semi-axis (green) is the mean squared displacement of all points from the median. The length σ_+ of the longer side of the asymmetric semi-axis (red) is the mean squared displacement of the points above the symmetric semi-axis, and the length σ_- of the shorter side of the asymmetric semi-axis (magenta) is the mean squared displacement of the points below the symmetric semi-axis.

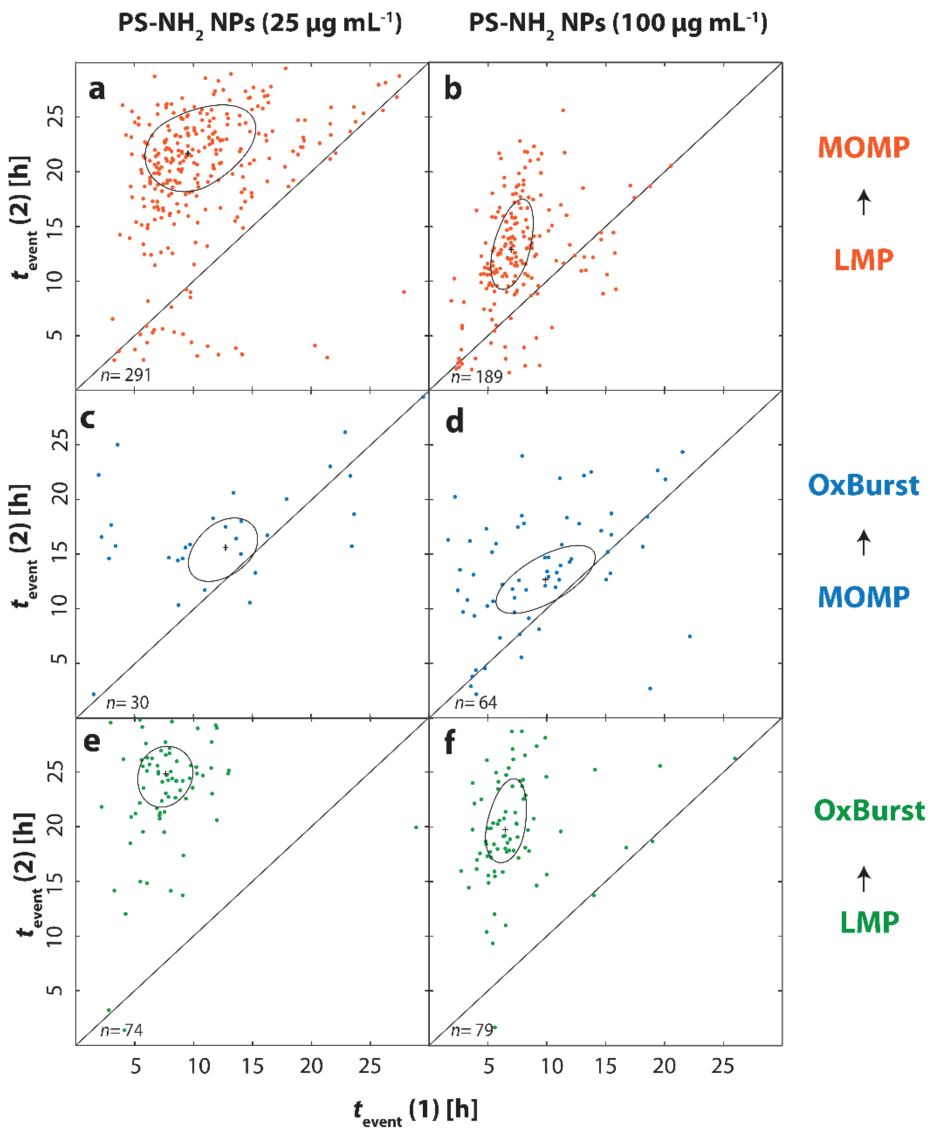

Supplementary Figure 5: Pairwise fluorescence marker correlations of single Huh7 cells. Scatter plots of event times t_{event} (1) and t_{event} (2) of marker pairs induced by exposure of cells to 25 µg mL^{-1} nanoparticles (left) and 100 µg mL^{-1} nanoparticles (right). The uniaxially asymmetric ellipses display the mean squared displacement of events points from the ellipse centre. MOMP was correlated with LMP (**Fig. 5a, b**) as well as with OxBurst (**Fig. 5c, d**). Additionally, LMP and OxBurst were correlated with each other (**Fig. 5e, f**). *n* is the number of cells shown in the respective scatter plot. The data was drawn from at least three experiments per marker combination. Detailed information on the Huh7 data is given in **Supplementary Table 7**.

Pearson correlation coefficient

In **Fig. 5**, the pearson correlation coefficient (PCC) of ROS production rate and event time of LMP or MOMP, respectively, is indicated.

The PCC was calculated using Matlab's function "corrcoef"[2]. Formally, the PCC of N points, each at coordinates (x_i, y_i) with $i = 1, \dots, N$, is given by:

$$\mathrm{PCC} = \frac{1}{N-1} \sum_{i=1}^{N} \frac{x_i - \mu_x}{\sigma_x} \frac{y_i - \mu_y}{\sigma_y}$$

Here, μ_x and μ_y are the mean, and σ_x and σ_y are the standard deviation of all coordinates x_i and y_i, respectively.

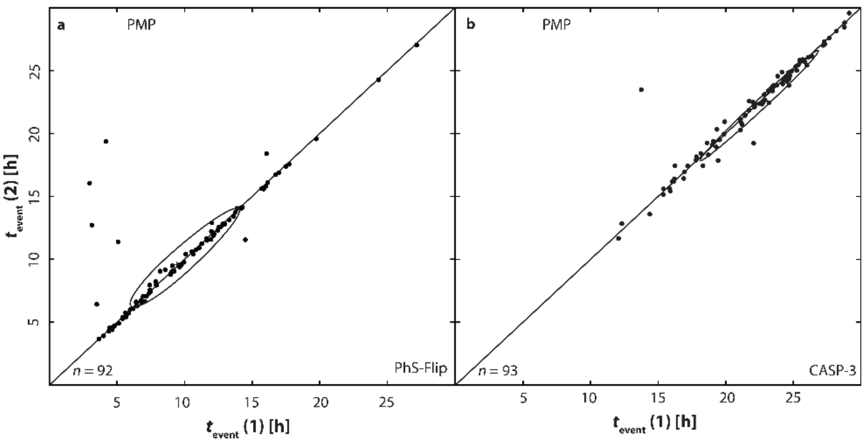

Supplementary Figure 6: Correlation of PMP with (**a**) PhS-Flip and (**b**) CASP-3. Cells were exposed to 25 µg mL^{-1} nanoparticles. *n* is the number of cells shown in the scatter plot. In both cases, the observed events occur simultaneously. Details of the cluster settings are given in **Supplementary Table 8**; the statistics in **Supplementary Table 9**. The exact positions of the cluster centres are given in **Supplementary Table 10**.

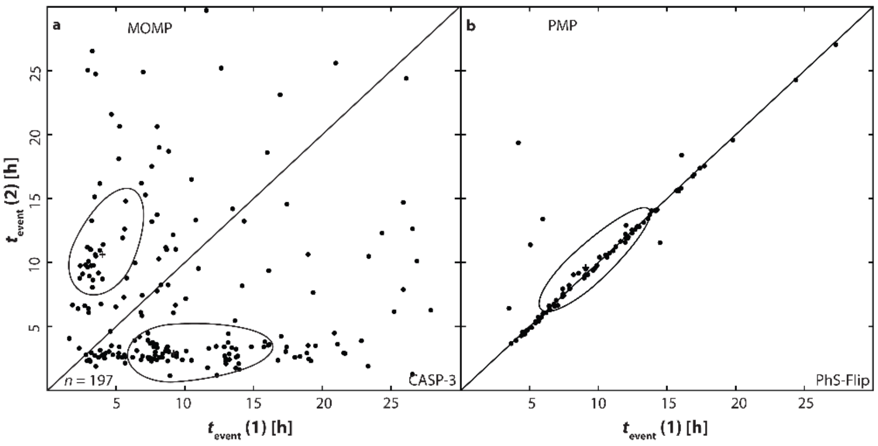

Supplementary Figure 7: Correlation of late markers. Single A549 cells after treatment with 100 µg mL^{-1} nanoparticles. *n* is the number of cells shown in the scatter plot. The correlation of the later event caspase-3 activation (CASP-3) with an earlier event (MOMP) (**a**) shows broader scattering, whereas PhS-Flip and PMP, the correlation of two late events, occur at the same time with a very narrow distribution (**b**). Details of the clustering settings are given in **Supplementary Table 8**, detailed information on the statistics in **Supplementary Table 11**, the exact positions of the cluster centres in **Supplementary Table 12**.

Supplementary Table 1: Low concentration of PS-NH_2 of 10 µg mL^{-1}.

First Marker	Second Marker	Cells with both signals
**MOMP**	OxBurst	16%
**LMP**	MOMP	4%
**LMP**	OxBurst	2%

Results of one experiment did not show any evident impact on A549 cells, indicated by a very low amount of cells showing both marker signals of early marker combinations. The results are comparable to the results of the negative control (**Supplementary Table 3**).

Supplementary Table 2: Overview of maxima of event-time distributions shown in **Figure 3**.

Marker	NP 25 µg mL^{-1} Nr. of cells	Maxima [h]	NP 100 µg mL^{-1} Nr. of cells	Maxima [h]	STS (2 µM) Nr. of cells	Maxima [h]
**LysoTracker**	892	12.9±0.18	1733	9.31±0.13	931	7.96±0.16
**TMRM**	1168	10.93±0.19	1144	7.81±0.17	1250	9.51±0.15
**CellROX**	1149	12.96±0.17	649	4.68±0.18	939	10.3±0.18
**Caspase-3/7**	231	20.59±0.3	470	8.75±0.26	252	15.0±0.4
**pSIVA**	75	16.2±0.8	102	7.9±0.5	81	13.9±0.8
**PI/ Toto-3 Iodide**	93	21.2±0.5	810	8.72±0.19	211	13.1±0.4

Maxima of the log-normal distributions fitted to the distributions of event times detected for each marker (NP = nanoparticles, STS = staurosporine).

Supplementary Table 3: Statistics of negative controls.

First Marker	Second Marker	Cells with both signals
MOMP	OxBurst	18%
LMP	MOMP	6%
LMP	OxBurst	17%
OxBurst	PMP	2%
LMP	PMP	0%
LMP	PhS-Flip	8%
PhS-Flip	PMP	0%
CASP	PMP	3%
PhS-Flip	MOMP	2%

A549 cells were only treated with two markers in Leibovitz's L15 medium, without any cell death trigger. Results in % are the sum of one to six experiments per marker combination, measured on different days. Each single experiment did not show more than 20% of cells with both marker signals.

Supplementary Table 4: Overview of statistics of **Figure 4**.

PS-NH$_2$ NP (25 µg mL^{-1})	Both	First only	Second only	None	Total
MOMP/OxBurst	726	305	90	95	1216
[%] of total	60				
LMP/MOMP	388	184	133	458	1163
[%] of total	33				
LMP/OxBurst	384	233	26	30	673
[%] of total	57				
**PS-NH$_2$ NP (100 µg mL^{-1})**	**Both**	**First only**	**Second only**	**None**	**Total**
MOMP/OxBurst	188	280	60	171	699
[%] of total	27				
LMP/MOMP	744	78	134	494	1450
[%] of total	51				
[%] of ellipse data in LMP-MOMP	56				
[%] of ellipse data in MOMP-LMP	42				
LMP/OxBurst	227	646	37	105	1015
[%] of total	22				
[%] of ellipse data in OxBurst-LMP	41				
[%] of ellipse data in LMP-OxBurst	34				
**STS (2 µM)**	**Both**	**First only**	**Second only**	**None**	**Total**
MOMP/OxBurst	481	274	62	82	899
[%] of total	54				
LMP/MOMP	580	170	510	258	1518
[%] of total	38				
[%] of ellipse data in LMP-MOMP	47				
[%] of ellipse data in MOMP-LMP	51				
LMP/OxBurst	235	325	223	129	912
[%] of total	26				
[%] of ellipse data in OxBurst-LMP	49				
[%] of ellipse data in LMP-OxBurst	35				
OxBurst/PMP (25 µg mL^{-1})	91	58	45	34	228
[%] of total	40				
OxBurst/PMP (100 µg mL^{-1})	234	87	421	134	876
[%] of total	27				
OxBurst/PMP (STS 2 µM)	139	481	113	408	1141
[%] of total	12				

The column "Both" gives the numbers of cells that showed clearly identifiable events for both markers, i.e., the cells shown in **Figure 4**. The column "First only" ("Second only") lists the numbers of cells for which only the marker plotted along the horizontal (vertical) axis showed an identifiable event. The column "None" contains the numbers of cells that did not show an identifiable signal for any marker. The column "Total" is the sum of analyzed cells (NP = nanoparticles, STS = staurosporine).

Supplementary Table 5: Delay times of scatter plots shown in **Figure 4**.

**NP 25 µg mL^{-1}**	**Centre of First Marker [h]**	**Centre of Second Marker [h]**	**Δ*t* [h]**	**Correlation Coefficient**	**Cluster Size**
MOMP/OxBurst	9.54±0.20	12.64±0.18	3.1±0.3	0.80	658
LMP/MOMP	16.2±0.3	20.30±0.28	4.1±0.5	0.77	387
LMP/OxBurst	13.83±0.25	17.87±0.25	4.0±0.4	0.72	347
**NP 100 µg mL^{-1}**	**Centre of First Marker [h]**	**Centre of Second Marker [h]**	**Δ*t* [h]**	**Correlation Coefficient**	**Cluster Size**
MOMP/OxBurst	3.98±0.08	5.27±0.16	1.29±0.18	0.46	147
MOMP/LMP	14.22±0.24	17.06±0.26	2.8±0.4	0.83	450
LMP/MOMP	11.18±0.27	13.9±0.4	2.7±0.5	0.81	291
LMP/OxBurst	4.94±0.16	9.0±0.5	4.0±0.6	0.23	68
OxBurst/LMP	5.21±0.19	6.8±0.5	1.6±0.6	0.60	48
**STS**	**Centre of First Marker [h]**	**Centre of Second Marker [h]**	**Δ*t* [h]**	**Correlation Coefficient**	**Cluster Size**
MOMP/OxBurst	8.73±0.16	12.69±0.17	3.96±0.24	0.56	461
MOMP/LMP	11.7±0.5	13.0±0.5	1.3±0.8	0.95	178
LMP/MOMP	10.58±0.18	15.41±0.29	4.8±0.4	0.39	402
LMP/OxBurst	8.26±0.28	19.4±0.5	11.2±0.6	-0.11	151
OxBurst/LMP	13.7±0.7	16.9±0.8	3.2±1.1	0.87	84
**OxBurst/PMP**	**Centre of First Marker [h]**	**Centre of Second Marker [h]**	**Δ*t* [h]**	**Correlation Coefficient**	**Cluster Size**
25 µg mL^{-1}	4.3±0.4	15.9±0.6	11.6±0.8	0.42	91
100 µg mL^{-1}	3.73±0.16	6.57±0.22	2.84±0.28	0.66	201
STS	5.45±0.25	15.7±0.4	10.2±0.5	0.38	135

For each exposure and marker combination, the positions of the cluster centers of the scatter plots in **Figure 4** are indicated, together with an error estimate. The “Centre of First Marker” is the centre of the cluster projected on the axis of the event mentioned first in the left column, and the “Centre of Second Marker” is the centre of the cluster projected on the axis of the event mentioned second. Δ*t* is the time difference between the first and the second event in hours, “Correlation Coefficient” the Pearson correlation coefficient of the events in the cluster and “Cluster Size” is the number of cells in the cluster (NP = nanoparticles, STS = staurosporine).

Supplementary Table 6: Mean shift clustering properties used for **Figure 4**.

NP 25 µg mL^{-1}	Diagonal border enforced	Kernel band width above diagonal [h]	Kernel band width under diagonal [h]
**MOMP/OxBurst**	no	4	
**LMP/MOMP**	no	6	
**LMP/OxBurst**	no	5	
**OxBurst/PMP**	no	9	
**NP 100 µg mL^{-1}**	**Diagonal border enforced**	**Kernel band width above diagonal [h]**	**Kernel band width under diagonal [h]**
**MOMP/OxBurst**	no	2	
**LMP/MOMP**	yes	5	5
**LMP/OxBurst**	yes	6	6
**OxBurst/PMP**	no	5	
**STS (2 µM)**	**Diagonal border enforced**	**Kernel band width above diagonal [h]**	**Kernel band width under diagonal [h]**
**MOMP/OxBurst**	no	3.5	
**LMP/MOMP**	yes	5	5
**LMP/OxBurst**	yes	9	8
**OxBurst/PMP**	no	5	

For each combination and inducer condition, the kernel bandwidth (in hours) used for mean shift clustering and the whether or not the diagonal of the scatter plots was treated as a cluster border are indicated. The diagonal was treated as cluster border when two clusters were expected. In these cases, clustering was performed separately for the data below and above the diagonal (NP = nanoparticles, STS = staurosporine).

Supplementary Table 7: Complete overview of Huh7 data (**Supplementary Fig. 5**).

NP (25 µg mL^{-1})\# of cells	Both	First only	Second only	None	Total
MOMP/OxBurst	30	96	22	85	233
[%] of total	13				
LMP/MOMP	291	333	62	66	752
[%] of total	39				
LMP/OxBurst	74	753	32	240	1099
[%] of total	7				
NP (100 µg mL^{-1})	Both	First only	Second only	None	Total
MOMP/OxBurst	64	136	58	185	443
[%] of total	14				
LMP/MOMP	189	226	45	230	690
[%] of total	27				
LMP/OxBurst	79	332	19	94	514
[%] of total	15				

NP 25 µg mL^{-1}	Centre of First Marker [h]	Centre of Second Marker [h]	Δt [h]	Correlation Coefficient	Cluster Size
MOMP/OxBurst	12.7±0.8	15.6±0.8	2.9±1.2	0.38	17
LMP/MOMP	9.5±0.3	21.67±0.24	12.2±0.4	0.39	264
LMP/OxBurst	7.59±0.29	24.9±0.4	17.3±0.5	0.12	65
NP 100 µg mL^{-1}	Centre of First Marker [h]	Centre of Second Marker [h]	Δt [h]	Correlation Coefficient	Cluster Size
MOMP/OxBurst	9.8±0.7	12.7±0.5	2.9±0.9	0.66	41
LMP/MOMP	6.93±0.15	12.9±0.4	6.0±0.5	0.47	155
LMP/OxBurst	6.48±0.21	19.8±0.5	13.3±0.6	0.32	69

NP 25 µg mL^{-1}	Diagonal border enforced	Kernel band width above diagonal [h]
MOMP/OxBurst	no	5.5
LMP/MOMP	no	4.5
LMP/OxBurst	no	3.5
NP 100 µg mL^{-1}	Diagonal border enforced	Kernel band width above diagonal [h]
MOMP/OxBurst	no	4
LMP/MOMP	no	3
LMP/OxBurst	no	4

The first table shows the statistics of the Huh7 data; see description of **Supplementary Table 4** for an explanation. The second table shows the delay times of the Huh7 scatter plots; see description of **Supplementary Table 5** for an explanation. The third table shows the mean-shift clustering properties for the Huh7 data; see description of **Supplementary Table 6** for an explanation.

Supplementary Table 8: Mean shift clustering properties of **Supplementary Figures 6 and 7**.

NP 25 µg mL^{-1}	Diagonal border enforced	Kernel band width above diagonal [h]	Kernel band width under diagonal [h]
**PhS-FLIP/PMP**	no	6.5	
**PMP/CASP-3**	no	6	

NP 100 µg mL^{-1}	Diagonal border enforced	Kernel band width above diagonal [h]	Kernel band width under diagonal [h]
**CASP-3/MOMP**	yes	5	5
**PhS-FLIP/PMP**	no	7	

The clustering settings are listed per marker combination and exposure. For detailed explanation, see description of **Supplementary Table 6**.

Supplementary Table 9: Statistics of **Supplementary Figure 6**.

Late Marker Combinations	Both	First only	Second only	None	Total
**PhS-FLIP/PMP (25 µg mL^{-1})**	92	5	163	155	415
**[%] of total**	22				
**PMP/CASP-3 (25 µg mL^{-1})**	93	46	31	255	425
**[%] of total**	22				

The column "Both" indicates the numbers and percentages of cells in which both markers detected an identifiable event. These are the cells depicted in **Supplementary Figure 6**. The columns "First only" and "Second only" indicate the numbers and percentages of cells for which only the event plotted on the horizontal or vertical axis, respectively, was identified. The column "None" indicates the numbers and percentages of cells for which no event was identified. The column "Total" indicates the total number of cells analysed (NP = nanoparticles, STS = staurosporine).

Supplementary Table 10: Delay times derived from the scatter plots shown in **Supplementary Figure 6**.

NP 25 µg mL^{-1}	Centre of First Marker [h]	Centre of Second Marker [h]	Δ*t* [h]
**PhS-FLIP/PMP**	9.4±0.5	9.6±0.5	0.2±0.8
**PMP/CASP-3**	22.6±0.5	22.9±0.5	0.3±0.8

The positions of the cluster centers of the scatter plots in **Supplementary Figure 6** are listed per event combination together with an error estimate. For detailed explanation, see description of **Supplementary Table 5**.

Supplementary Table 11: Statistics of **Supplementary Figure 7**.

Late Marker Combinations	Both	First only	Second only	None	Total
CASP-3/MOMP (100 μg mL^{-1})	87	3	158	7	255
[%] of total	34				
PhS-FLIP/PMP (100 μg mL^{-1})	197	207	37	45	486
[%] of total	41				

For details, see description of **Supplementary Table 4**.

Supplementary Table 12: Delay times derived from the scatter plots shown in **Supplementary Figure 7**.

**NP 100 µg mL^{-1}**	**Centre of First Marker [h]**	**Centre of Second Marker [h]**	***Δt* [h]**
**CASP-3/MOMP**	4.0±0.4	10.6±0.6	6.6±0.8
**MOMP/CASP-3**	2.92±0.21	9.2±0.6	6.3±0.7
**[%] of ellipse data in CASP-3-MOMP**	41		
**[%] of ellipse data in MOMP-CASP-3**	52		
**PhS-FLIP/PMP**	9.1±0.5	9.6±0.5	0.5±0.8

The positions of the cluster centers of the scatter plots in **Supplementary Figure 7** are listed per exposure and event combination. For details, see description of **Supplementary Table 5.**

References:

1. Raue, A. *et al.* Lessons Learned from Quantitative Dynamical Modeling in Systems Biology. *PLoS One* **8,** (2013).
2. MathWorks, T. MATLAB 2017a,. (2017).
3. Cheng, Y. Mean Shift, Mode Seeking, and Clustering. *IEEE Trans. Pattern Anal. Mach. Intell.* **17,** 790–799 (1995).
