# Supplementary material for: A high-throughput microscopy method for single-cell analysis of event-time correlations in nanoparticle-induced cell death
Source: Commun Biol. 2019 Jan 24;2:35. doi: 10.1038/s42003-019-0282-0 (PMC6345847; doi:10.1038/s42003-019-0282-0)
Supplement: Supplementary file 2 — Description of Additional Supplementary Files [file 42003_2019_282_MOESM2_ESM.pdf]

Supplementary Movie 1: Single-cell array with CellROX and PI staining. An example of a time-lapse movie after exposure of a single-cell array to 2 µM STS. Cellular stress is illustrated by the CellROX (purple), and cell death by the PI (yellow) fluorescence. Scale bar: 300 µm.

Supplementary Movie 2: Single cells with CellROX and LysoTracker staining. A movie showing the behaviour of four single cells after administration of 25 µg mL^{-1} PS-NH_2 nanoparticles. CellROX (purple) and LysoTracker (cyan) were used to visualize intracellular stress and lysosomal breakdown. Scale bar: 30 µm.

Supplementary Movie 3: Time-lapse sequence of 30 h of A549 cells, undergoing cell death, with the corresponding time traces. Selection of exemplary single A549 cells, treated with 100 µg mL^{-1} PS-NH_2 nanoparticles, out of one field of view in phase contrast, with CellROX or LysoTracker staining. Scale bar: 30 µm.
